# Association between organ dysfunction during ICU stay and post-intensive care syndrome at 1 year after ICU discharge: a prospective cohort study

**DOI:** 10.1186/s40560-026-00885-4

**Published:** 2026-05-28

**Authors:** Mahan Sadjadi, Matteo Marcello, Miles Drammeh, Hendrik Booke, Katarzyna Hognon-Bak, Lisa Aistleitner, Ludwig Maximilian Schöne, Christian Strauß, Moritz J. Mertes, Alexander Zarbock

**Affiliations:** 1 Department of Anesthesiology, Intensive Care and Pain Medicine, University Hospital Münster, Albert-Schweitzer-Campus 1, Geb. A1, 48149 Münster, Germany; 2 Department of Nephrology, Dialysis and Transplantation, San Bortolo Hospital, Vicenza, Italy; 3 Department of Anesthesiology and Intensive Care, St. Raphael Hospital, Krakow, Poland

**Keywords:** PICS, Post-intensive care syndrome, Critical illness, Intensive care, Acute kidney injury, Long-term outcomes, Organ dysfunction

## Abstract

**Background:**

Post-Intensive Care Syndrome (PICS) affects a substantial proportion of intensive care unit (ICU) survivors. This study investigates whether granular features of the ICU course including organ dysfunction are associated with the prevalence of PICS one year after ICU discharge.

**Methods:**

This was a prospective single-center cohort study of 159 adult surgical ICU survivors enrolled between September 2023 and August 2024. Standardized assessments for PICS domains were performed at 3 and 12 months; PICS was defined a priori as impairment in ≥ 2 of 3 domains (physical, cognitive, psychological). ICU exposure data were ascertained retrospectively from electronic health records. We compared baseline, illness severity and ICU-course variables between patients with and without PICS at 12 months. The primary exposure was the burden of critical illness, operationalized as the sum of organ dysfunctions during the ICU stay. Discriminative ability was evaluated across the organ dysfunction count model, a data-driven three-variable model as well as baseline-only and severity-score models as comparators.

**Results:**

At 12 months, 49 of 159 patients (31%) met PICS criteria. Baseline demographics and comorbidities did not differ between groups. Patients with PICS had longer and more complicated ICU stays, and more organ dysfunctions. Each additional organ dysfunction was associated with significantly higher odds of PICS (odds ratio [OR] 2.91; bias-corrected and accelerated [BCa] bootstrap 95% CI 1.92–6.00; *p* < 0.001). The organ dysfunction count demonstrated good discrimination (area under the receiver operating characteristic curve [AUC] 0.83; 95% CI 0.75–0.91; optimism-corrected AUC 0.82), significantly exceeding a baseline-only model containing demographics and comorbidities (AUC 0.60; 95% CI 0.50–0.71; DeLong *p* < 0.001), as well as outperforming admission Sequential Organ Failure Assessment (SOFA, AUC 0.63), and Acute Physiology And Chronic Health Evaluation II (APACHE II, AUC 0.64) models. An exploratory, data-driven three-variable model yielded an apparent AUC of 0.87 (optimism-corrected 0.83) with borderline calibration.

**Conclusions:**

In this exploratory analysis, the breadth of organ system involvement during critical illness, captured by the number of organ dysfunctions, was more strongly associated with PICS at 12 months after ICU discharge and demonstrated greater discriminative ability than any baseline demographics or comorbidities as well as SOFA and APACHE scores. These findings are hypothesis-generating and require external validation before clinical application.

**Supplementary Information:**

The online version contains supplementary material available at 10.1186/s40560-026-00885-4.

## Background

Survivors of critical illness often face prolonged and complex recovery trajectories after intensive care [1–3]. A substantial proportion experiences persistent physical, cognitive and psychological impairments, collectively termed Post-Intensive Care Syndrome (PICS) [4–6]. Reported 12-month prevalence of PICS varies by population and definition, and a range of variables have been described as risk factors [7–10]. However, much of the literature relies on proxy measures of illness severity, available metrics are restricted to few time points, and granular ICU data remains scarce, making it difficult to disentangle the relative contribution of complications and organ dysfunctions during ICU stay on the one hand from baseline vulnerability on the other [11]. Moreover, few studies have evaluated metrics from the ICU trajectory that could be systematically tested for discrimination, and be implemented in the clinical setting to aid follow-up planning or resource allocation following critical illness [11–13]. Understanding which factors drive the development of PICS is essential. If specific in-ICU features, organ dysfunctions like acute kidney injury (AKI), complications like prolonged hypotension, or therapeutic measures like extended ventilation are independently predictive, these could represent modifiable targets for prevention. Conversely, if post-ICU morbidity is largely determined by fixed baseline traits, interventional efforts would need to shift toward prehabilitation or post-discharge rehabilitation. Identifying patients at highest risk for PICS at or around ICU discharge could enable targeted follow-up and early intervention.

Multiple prior studies have identified organ dysfunctions including delirium [14], AKI [15, 16] and Acute Respiratory Distress Syndrome (ARDS) [17, 18] as risk factors for long-term impairment after critical illness. However, these have typically been examined in isolation, and the cumulative breadth of organ system involvement across the ICU stay has not been formally evaluated as a composite metric for PICS risk. The biological rationale for such an approach rests on the observation that critical illness frequently involves multi-system physiological derangement, and that each additional organ dysfunction may compound the systemic inflammatory, endothelial, and neurohumoral insult that drives long-term sequelae [15, 16, 19, 20]. To address this knowledge gap, we conducted a prospective observational cohort study of adult ICU survivors with a standardized 1-year follow-up. We hypothesized that the cumulative number of organ dysfunctions during the ICU stay, reflecting what can be conceptualized as the burden of injury, would be more strongly associated with PICS at 12 months than baseline demographics, comorbidity profiles or clinical scores at ICU admission or at any single timepoint during the ICU course. Specifically, we evaluated a pragmatic organ dysfunction metric, defined as the number of organ systems exhibiting dysfunction at any point during the ICU stay, and tested its association with the 12-month prevalence of PICS. In addition, we explored whether a small set of clinically plausible ICU-course variables could incrementally improve discrimination beyond the organ dysfunction count. We compared the discriminative ability of the organ dysfunction count with that of a baseline-only model containing demographic and comorbidity variables, as well as with established severity scores (admission Sequential Organ Failure Assessment [SOFA], admission Acute Physiology and Chronic Health Evaluation [APACHE II], and most-severe SOFA). Our aim was to provide a pragmatic risk signal that can be derived from routinely documented ICU data at discharge, and an exploratory multivariable benchmark to inform validation studies and allow for the generation of hypotheses that would help the future design of targeted interventions.

## Methods

### Study design and setting

This was a prospective single-center cohort study conducted across five surgical ICUs of a tertiary academic hospital in Münster, Germany. Prospective, standardized outcome assessments were conducted at 3 and 12 months post-ICU discharge. ICU exposure and baseline data were ascertained retrospectively from detailed electronic health records and structured interviews for all enrolled participants. Ethics approval was obtained from the Research Ethics Committee of the Chamber of Physicians, Westfalen-Lippe, and the University of Münster (2023-180-f-S). Written informed consent was obtained from all participants. Reporting follows STROBE [21] guidelines.

### Participants

**Fig 1:**
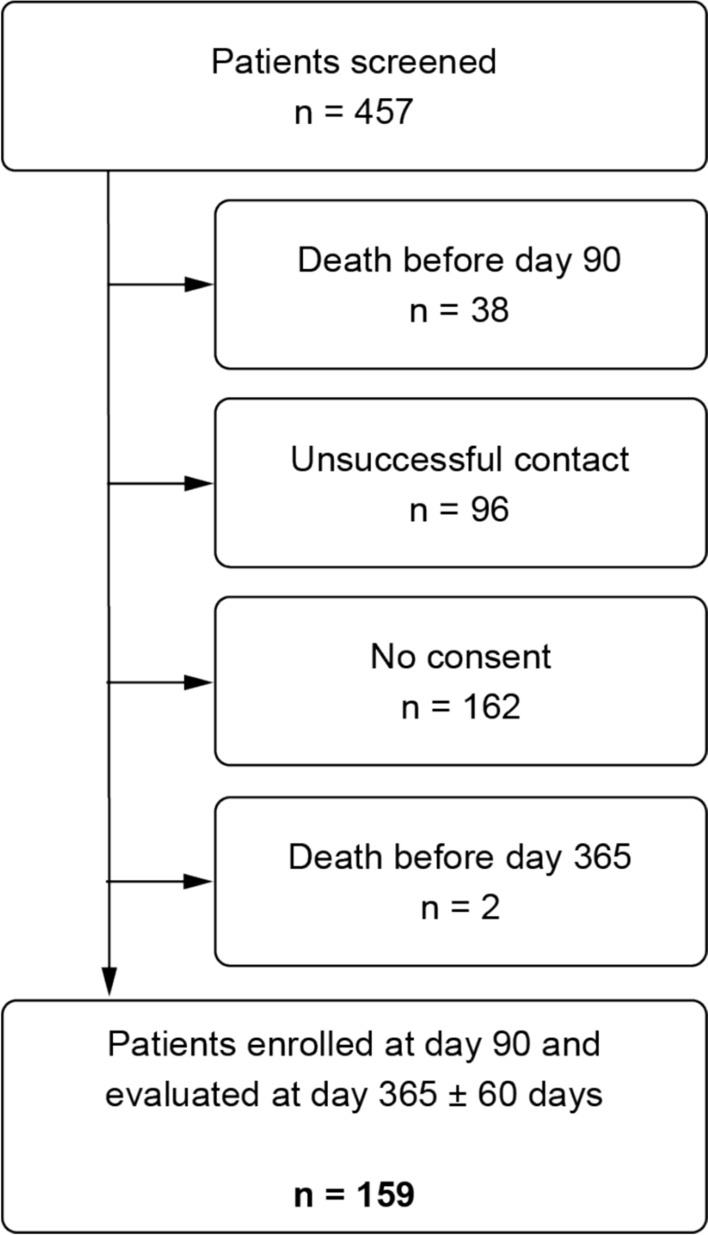
Study flowchart

Eligible patients were adults (≥ 18 years) who survived an index ICU admission of 72 h or longer between 1 September 2023 and 31 August 2024. Screening and recruitment into the study cohort occurred at 90 days after ICU discharge. All participants were required to give written consent. Reasons for exclusion are summarized in the study flowchart (Fig. 1).

### Procedures and measurements

Baseline and ICU data were collected by hand for each participant. Baseline functional status was reconstructed through structured patient interviews at the 3-month visit, review of pre-admission medical records including primary care and specialist documentation, and, where available, corroboration by family members. Patients with documented pre-existing diagnoses of dementia, depression, anxiety disorder, or chronic mobility impairment were identified, and their PICS domain classification was interpreted in the context of this information. However, formal validated pre-ICU assessments of cognitive, physical, and psychological function were not performed. Baseline data included age, sex, BMI and pre-existing conditions. ICU exposures were recorded by organ system and included durations of invasive procedures, complications (e.g. bleeding, infection) and organ-support-free days (e.g. ventilation-, vasopressor-, and nasogastric-tube-feeding-free). We prespecified a pragmatic organ dysfunction count as the sum (0–5) of five binary variables during the ICU stay, with one indicator per organ system:

1. Neurologic dysfunction–delirium: assessed using the confusion assessment method for the ICU (CAM-ICU) performed at protocolized sedation interruptions, supplemented by clinical documentation of neurologic status. Delirium was chosen because of its established association with long-term cognitive impairment, a core PICS domain. The Glasgow Coma Scale (GCS) was not used because it lacks specificity for intrinsic brain dysfunction. We acknowledge that comatose patients cannot be assessed with CAM-ICU and may be misclassified as delirium-negative; persistent coma unrelated to sedation was rare in our cohort.
2. Respiratory dysfunction–moderate or severe hypoxemia: Defined as PaO2/FiO2 ratio < 200 or paO2 < 60 mmHg on two consecutive arterial blood gas measurements. Milder thresholds were not used to retain specificity because transient mild hypoxemia is ubiquitous in postoperative ventilated patients.
3. Circulatory dysfunction–hypotension: defined as occurrence of mean arterial pressure (MAP) < 65 mmHg sustained for > 10 min.
4. Renal dysfunction–acute kidney injury: defined using Kidney Disease: Improving Global Outcomes (KDIGO) consensus criteria [22], which capture creatinine trajectories and urine output, providing greater sensitivity and specificity than absolute creatinine thresholds alone.
5. Hepatic dysfunction: defined as daily hepatic SOFA subscore ≥ 2 [23]).

The organ-level criteria were selected for their clinical sensitivity and relevance in the surgical ICU population. The cumulative nature of the organ dysfunction count-integrating whether each organ system was affected at any point during the ICU stay-captures temporal information that single-timepoint aggregates like the SOFA score cannot reflect. [23, 24]. Each organ dysfunction was scored as present (1) or absent (0) based on whether the specified criterion was met at any point during the index ICU admission. The organ dysfunction count thus represents the cumulative number of organ systems affected over the entire ICU course.

### Outcome measures

The primary outcome was PICS at 12 months post-ICU discharge, defined a priori as impairment in ≥ 2 of 3 domains (physical, cognitive, psychological). Outcome assessment was performed at 12 ± 2 months post-ICU discharge for all patients.

Outcome domains were assessed by verified measures as proposed by consensus guidelines and definitions [6, 25]. Physical function was assessed by patient reporting using the mobility item of the European Quality of Life 5 Dimensions 5 Level Version (EQ5D5L) questionnaire [6]. In addition, verified measures of Timed Up-and-Go and Handgrip Strength tests were used as previously recommended [25]. The physical domain was considered impaired if any measure met guideline impairment thresholds.

Cognitive function was assessed using the Montreal Cognitive Assessment (MoCA) with a recommended education correction. Impairment was defined as MoCA < 26 [6]. This was supplemented with the Animal Naming Test (ANT). Impairment was defined as ANT < 14 [25]. The Cognitive domain was considered impaired if either test met the impairment threshold.

Psychological status was defined as impaired as follows: For depression, the Patient Health Questionnaire-8 (PHQ-8) was used and impairment was defined as a test score ≥ 10 [25]. For anxiety, impairment was defined as a test score ≥ 10 of the Generalized Anxiety Disorder Scale-7 (GAD-7) [25]. For Post Traumatic Stress Disorder (PTSD) symptoms we used the PTSD checklist for the Diagnostic and Statistical Manual of Mental Disorders 5 (PCL-5) over Impact of Event Scale-Revised (IES-R) per recent recommendations and DSM-5 criteria alignment [26]. Impairment was defined as a test score ≥ 33. Psychological domain function was considered impaired if any instrument met the above-described threshold. In addition to the above analysis of PICS, health-related quality of life at 12 months was determined via EQ-5D-5L (German value set) with index scores derived per EuroQol guidelines [27]. Baseline status could only be assessed post-hoc but this was actively pursued in as much detail as possible by patient recall and extensive examination of electronic health records.

### Statistical analysis

Summary statistics are reported as mean (SD) or median (IQR) as appropriate, or as counts (%). For between-group comparisons (PICS vs no PICS) we used Welch’s *t*-test or Mann–Whitney U for continuous variables, depending on variable characteristics, and Chi-square or Fisher’s exact for categorical variables. Effect sizes are reported as mean or Hodges-Lehmann median differences with 95% CIs, and odds ratios with 95% CIs. The primary predictive analysis is a univariable binary logistic regression with the number of organ dysfunctions as the sole predictor of PICS; results are reported as odds ratio per additional dysfunction (95% CI). For the exploratory ICU‑course model we prespecified a candidate set of eight variables based on biological plausibility: AKI (yes/no), acute liver failure (yes/no), hypoxemia (yes/no), delirium (yes/no), ICU‑acquired infection (yes/no), vasopressor‑free days in 30 days (0–30), ventilation‑free days in 30 days (0–30), and duration of the index surgical procedure (minutes). To avoid double counting and collinearity, we did not include more than one variable for each organ system, and we omitted ICU length of stay and nasogastric‑feeding‑free days when ‘free‑days’ metrics were considered. In the forward selection (likelihood ratio) variables were entered at p < 0.05 and removed at p > 0.10, with a maximum of ≤ 3 predictors to limit events‑per‑variable. Age and sex were forced in a prespecified sensitivity analysis. Model performance was rated regarding discriminatory ability using area under the ROC (receiver operating characteristic) curve (AUC) with 95% CI. Calibration was assessed with Hosmer–Lemeshow goodness-of-fit and overall accuracy was explored with the Brier score (mean squared error of predicted probabilities). Since the cohort size was too small to be split for confirmatory analyses, internal validation was done using bootstrap resampling (1000 samples). A linear regression of EQ-5D-5L index on the number of organ dysfunctions was prespecified as a secondary analysis. The association between organ dysfunction count and health-related quality of life (EQ-5D-5L index) at 12 months was assessed using both univariable and multivariable linear regression. The multivariable model was adjusted for age, sex, and pre-existing comorbidities (hypertension, diabetes, chronic kidney disease [CKD], chronic obstructive pulmonary disease [COPD], depression, cancer). The influence of other individual exposures on the PICS outcome is explored in univariate analyses.

Predictor missingness was minimal (≤ 4 records per variable). Primary regression analyses (logistic for PICS and linear for EQ‑5D‑5L) were complete‑case. Given the low level of predictor missingness, we did not perform multiple imputation. Patients who died before day 90 or did not consent were not included in regression analyses per ethics approval.

To formally test the comparative hypothesis of ICU-course variables offering better discrimination than baseline factors, we constructed a baseline-only logistic regression model including age, sex, body mass index (BMI), and pre-existing comorbidities (hypertension, diabetes mellitus, CKD, COPD, depression, and cancer) as predictors of PICS at 12 months. The discriminative performance (AUC) of this model was compared to the organ dysfunction count model and to a combined model including both baseline factors and the organ dysfunction count using DeLong's test for correlated ROC curves. AUC values and Youden’s J-derived cutoffs are reported as exploratory assessments of discriminative ability and should not be interpreted as validated prediction tools.

Given the modest size of our cohort, all analyses were planned as exploratory, even if the analysis plan for the primary outcome and key secondary outcome was prespecified. *P* values are descriptive. All tests were two-sided with α = 0.05. Statistical analyses were conducted using IBM SPSS Statistics (version 29.0; IBM, Armonk, NY, USA) and R (version 4.5.2; R Foundation for Statistical Computing, Vienna, Austria).

## Results

### Cohort and follow-up

Between September 2023 and August 2024, 159 ICU survivors were enrolled at 90 days post-ICU discharge and completed the 12-month PICS assessment (Fig. 1). At 12 months, 49/159 (31%) met the predefined PICS criteria. Fifty patients (32%) had psychological symptoms, 46 (29%) experienced cognitive impairment, and 49 (31%) had muscular weakness. Baseline demographics and comorbidities like hypertension, diabetes, CKD, COPD, depression, cerebrovascular disease, and cancer did not differ between the groups. (Table 1).

**Table 1:** Differences between patients with and without PICS at 12 months

Characteristic	No PICS (*n* = 110)	PICS (*n* = 49)	Effect size	*P* value
Age, median (IQR)	62 (44.5–71)	59 (52.5–70)	− 0.5 (− 6 to 4)^a^	0.81
Male sex, *n* (%)	83 (75.5)	33 (67.3)	1.5 (0.7–3.1)^b^	0.29
BMI, mean (SD)	27.8 (6.2)	27.7 (5.4)	− 0.1 (-2.4–2.2)^c^	0.93
Hypertension, *n* (%)	64 (58.2)	29 (59.2)	1.0 (0.5–2.4)^b^	0.87
Myocardial infarction, *n* (%)	12 (10.9)	5 (10.6), 2 missing	1.1 (0.3–3.8)^b^	0.91
Congestive heart failure, *n* (%)	20 (18.2)	9 (18.4)	1.0 (0.3–2.7)^b^	0.93
Peripheral artery disease, *n* (%)	19 (17.4), 1 missing	8 (17.0), 2 missing	1.0 (0.3–2.7)^b^	0.93
Depression, *n* (%)	9 (8.2)	5 (10.6), 2 missing	1.4 (0.4–5.2)^b^	0.60
Cerebrovascular disease, *n* (%)	8 (7.3)	3 (6.1)	0.8 (0.2–4.1)^b^	0.78
Chronic obstructive pulmonary disease, *n* (%)	8 (7.3)	4 (8.2)	1.2 (0.3–5.2)	0.79
Diabetes, *n* (%)	25 (22.7)	8 (17.0), 2 missing	0.7 (0.3–2.0)^b^	0.50
CKD stage 3a or worse, *n* (%)	9 (8,2)	2 (4,1)	0.48 (0.1–2.3)^b^	0.36
Cancer (solid tumor), *n* (%)	19 (17.3)	10 (20.4)	1.3 (0.5–3.4)^b^	0.67
APACHE II score at ICU admission, mean (SD)	18.2 (7.2)	21.9 (7.1)	3.7 (0.6–6.9)^c^	0.02
SOFA score at ICU admission, mean (SD)	6 (3.3)	8 (3.5)	2.1 (0.6–3.5)^c^	0.007
Hospital length of stay (days), median (IQR)	18 (12 – 31)	25.5 (17 – 35)	6 (2–11)^a^	0.012
Duration of surgery (min), mean (SD)	225 (129)	341 (155)	117 (50–184)^c^	0.001
Vasopressor-free days in 30 days, median (IQR)	26 (23–28.5)	22 (10–25.25)	− 5 (− 8 to − 3)^a^	< 0.001
Ventilation-free days in 30 days, median (IQR)	29 (28–30)	26 (21–28)	− 2 (− 4 to − 1)^a^	< 0.001
Nasogastric-tube-free days in 30 days, median (IQR)	30 (28–30)	24 (6.75–30)	− 3 (− 8 to 0)^a^	< 0.001
ICU length of stay (days), median (IQR)	9 (6–15)	16 (11.25–24)	7 (3–9)^a^	< 0.001
Organ dysfunctions during ICU stay				
Delirium during ICU, *n* (%)	22 (20)	26 (53.1)	4.5 (1.8–10.9)^b^	< 0.001
Psychoactive drugs during ICU, *n* (%)	74 (67.3)	46 (93.9)	7.2 (1.6–32.7)^b^	0.004
Hypoxemia during ICU (%)	41 (37.3)	36 (75), 1 missing	5.1 (2.0–13.0)^b^	< 0.001
Hypotension during ICU, *n* (%)	77 (70.0)	46 (93.9)	6.3 (1.4–28.9)^b^	0.008
ECMO during ICU, *n* (%)	5 (4.5)	7 (15.6), 4 missing	4.4 (1.0–19.6)^b^	0.038
Acute kidney injury during ICU, *n* (%)	39 (35.5)	41 (85.4), 1 missing	9.8 (3.4–28.4)^b^	< 0.001
CRRT during ICU, *n* (%)	3 (2.7)	10 (21.7), 2 missing	10.1 (2.0–51.8)^b^	0.001
Glucose dysregulation during ICU, *n* (%)	69 (62.7)	42 (85.7)	3.2 (1.1–9.2)^b^	0.029
Infection during ICU, *n* (%)	40 (36.4)	32 (65.3)	3.3 (1.4–7.9)^b^	0.006
Atrial fibrillation during ICU, *n* (%)	28 (25.5)	13 (27.7), 2 missing	1.1 (0.–2.9)^b^	0.79
Acute liver injury during ICU, *n* (%)	16 (14.5)	19 (38.8)	3.6 (1.4–9.5)^b^	0.007
≥ 3 Organ dysfunctions, *n* (%)	29 (26.4)	41 (83.7)	13.8 (4.7–40.8)^b^	< 0.001

PICS is defined as co-occurrence of symptoms in at least 2 of 3 PICS domains (cognition, psyche, motor function). Exploratory analyses. No adjustment for multiplicity. a, Hodges–Lehmann median difference (PICS − No PICS) with 95% CI from Mann–Whitney U. b, odds ratio (PICS vs No PICS) with 95% CI; p from Fisher’s exact if any expected cell < 5, else Pearson chi-square. c, mean difference (PICS − No PICS) with 95% CI from Welch’s *t*-test. *P*-values represent descriptive between-group comparisons. Effect sizes and confidence intervals should be prioritized for interpretation

### ICU course and breadth of organ system involvement

Organ dysfunction and complications were significantly more frequent in patients with PICS. AKI occurred in 85% of cases with PICS and 36% in patients without PICS (odds ratio [OR] 9.8; 95% CI 3.4–28.4). Acute liver injury [39 vs 15% (OR 3.6; 95% CI 1.4–9.5)], delirium [53% vs 20% (OR 4.5; 95% CI 1.8–10.9)], ICU-acquired infection (65 vs 36% (OR 3.3; 95% CI 1.4–7.9), hypotension [94% vs 70% (OR 6.3; 95% CI 1.4–28.9)], and hypoxemia [75% vs 37% (OR 5.1; 95% CI 2.0–13.0)], too, were significantly more frequent in patients with PICS compared to patients without PICS. Patients with PICS had a longer length of ICU [16 (11–24) vs 9 (6–15) days; median difference 7, 95% CI 3–9] and hospital stay [25.5 (17–35) vs 18 (12–31) days; median difference 6, 95% CI 2–11] compared to patients without PICS. They also had fewer support-free days in the first 30 days: vasopressor-free [22 (10–25) vs 26 (23–28.5) days; median difference − 5, 95% CI − 8 to – 3], ventilation-free [26 (21–28) vs 29 (28–30) days; median difference − 2, 95% CI − 4 to − 1], and nasogastric-feeding-free [24 (6.75–30) vs 30 (28–30) days; median difference − 3, 95% CI − 8 to 0]. Supplementary Table 3 presents patient characteristics stratified by organ dysfunction count (0–2 vs. ≥ 3), demonstrating that baseline demographics and comorbidities were similar across exposure groups while PICS prevalence differed markedly (9.0 vs. 58.6%).

### Organ dysfunction as a predictor of PICS

**Fig 2:**
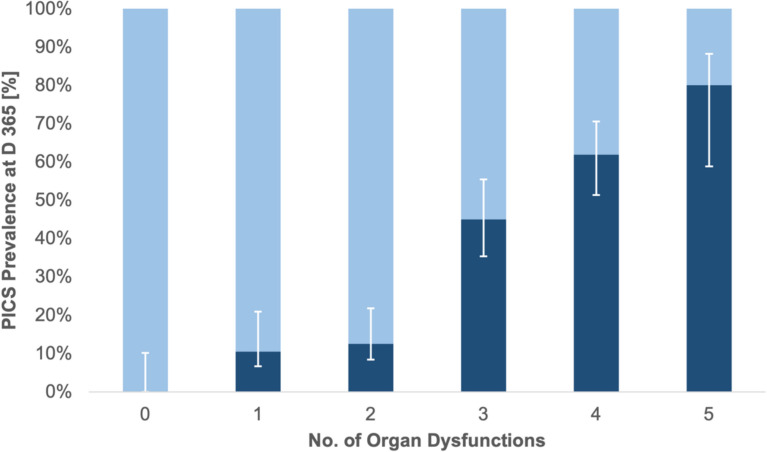
PICS at 12 months by No. of Organ Dysfunctions

The organ dysfunction score showed a strong relationship with the occurrence of PICS (Fig. 2). In patients with zero to two dysfunctions, PICS prevalence at 12 months was only 9%; in patients with three or more dysfunctions, the prevalence was 58.6% (OR 13.8; 95% CI 4.7–40.8). Each additional organ dysfunction was associated with a 2.91-fold higher odds of PICS (OR 2.91; Bca-Bootstrap 95% CI 1.92–6.00; p < 0.001). In AUC analyses, the number of organ dysfunctions demonstrated good discriminative ability for PICS. The apparent AUC was 0.83 (95% CI 0.75–0.91); bootstrap internal validation (1000 resamples) estimated optimism at 0.01, yielding an optimism-corrected AUC of 0.82 with a calibration slope of 0.96 and intercept of − 0.02, indicating good calibration stability and minimal overfitting (Supplementary Fig. 1). The Hosmer–Lemeshow test confirmed adequate calibration (*χ*^2^ = 0.996; *p* = 0.910; Brier score 0.147). The optimal cutoff determined by using the Youden’s J was an organ dysfunction score ≥ 3 (sensitivity 0.84, specificity 0.73). A more specific threshold of ≥ 4 had sensitivity 0.55 and specificity 0.88. This threshold is data-derived and requires external validation.

### Comparison with baseline characteristics

A baseline-only logistic regression model including age, sex, BMI, and pre-existing comorbidities (hypertension, diabetes, CKD, COPD, depression, cancer) yielded an AUC of 0.60 (95% CI 0.50–0.71), confirming that baseline patient characteristics had no discriminative value for 12-month PICS. The overall model was not significant (Omnibus Chi-square = 6.229,* df* = 9, *p *= 0.72), and no individual baseline variable reached statistical significance (all *p* > 0.10). As a formal comparison, the organ dysfunction count model demonstrated significantly greater discrimination than the baseline-only model (AUC 0.82 vs. 0.60; DeLong test *Z* = 4.13; *p* < 0.001).

A combined model including both baseline factors and the organ dysfunction count yielded an AUC of 0.82 (95% CI 0.72–0.91), identical to the organ dysfunction count model alone (DeLong test *Z* = 0.41; *p* = 0.68). In the combined model, the organ dysfunction count remained the dominant predictor (adjusted OR 2.81 per additional dysfunction; 95% CI 1.81–4.36; *p* < 0.001), while no individual baseline characteristic reached statistical significance (all *p* > 0.40).

### Exploratory three-variable model

In an exploratory forward-selected logistic regression model constrained to three predictors, AKI during ICU (adjusted OR 6.82; 95% CI 1.94–23.99; *p* = 0.003), longer duration of index surgery (adjusted OR 1.004 per minute; 95% CI 1.001–1.008; *p* = 0.026), and fewer vasopressor-free days (adjusted OR 0.89 per day; 95% CI 0.82–0.97; *p* = 0.011) were independently associated with PICS at 12 months. The apparent AUC for the exploratory three-variable model was 0.87 (95% CI 0.79–0.95); estimated optimism was 0.042, yielding an optimism-corrected AUC of 0.83. Across 1000 bootstrap resamples, the inclusion frequency was 92% for AKI, 69% for vasopressor-free days, and 60% for duration of index surgery. The remaining five candidate variables were selected in fewer than 30% of resamples each (Supplementary Table 2). Bootstrap analyses showed evidence of overfitting (optimism-corrected calibration slope 0.80; intercept 0.07) (Supplementary Fig. 2). The lower calibration slope for the exploratory model indicates that its predicted probabilities are too extreme, overestimating risk for high risk patients and underestimating risk for low-risk patients, consistent with the borderline Hosmer–Lemeshow result (*p* = 0.049). These findings illustrate the hypothesis-generating nature of this work and are not intended for clinical implementation.

### Quality of life

In univariable linear regression, each additional organ dysfunction was associated with a lower EQ-5D-5L index at 12 months (unadjusted *β* = − 0.052; 95% CI − 0.070 to − 0.034; *p* < 0.001; *R*^2^ = 0.233). After adjustment for age, sex, and key comorbidities, the association remained significant (adjusted *β* = − 0.044 per additional organ dysfunction; 95% CI − 0.068 to − 0.020; *p* < 0.001; adjusted *R*^2^ = 0.22). No baseline characteristic was significantly associated with EQ-5D-5L index in the adjusted model. For clinical context, a patient with four organ dysfunctions versus zero would be expected to have an EQ-5D-5L index approximately 0.18 points lower, which exceeds the commonly cited minimally important difference of 0.04–0.08 for this instrument.

### Sensitivity and robustness

**Fig. 3:**
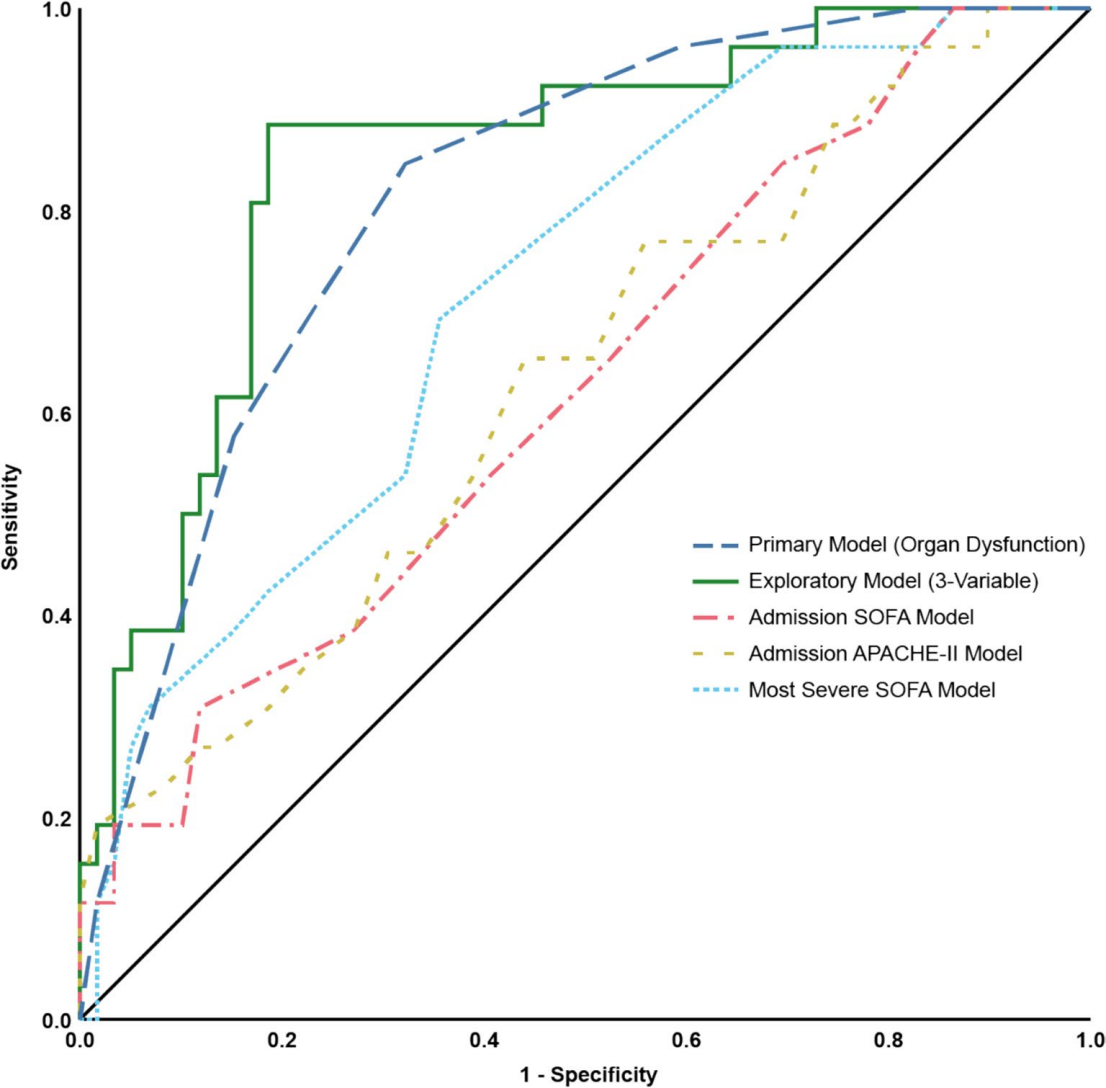
Receiver operating characteristic curves for prediction of PICS at 12 months

Severity scores (APACHE II, SOFA) performed worse regarding association with 12-month PICS than the organ dysfunction and exploratory models: admission SOFA AUC was 0.63 (95% CI 0.51–0.76), admission APACHE II AUC was 0.64 (0.52–0.76), most-severe SOFA AUC was 0.71 (0.61–0.79), and ICU-discharge SOFA AUC was 0.56 (0.45–0.67). All were substantially below the organ dysfunction count (AUC 0.82) and the three-variable model (AUC 0.83) (Table 2). Performance of models is presented graphically in Fig. 3.

**Table 2:** Logistic regression models and model characteristics for PICS at 12 months

Model	Model components	Performance
Baseline	Age, sex, BMI, comorbidities (hypertension, diabetes, CKD, COPD, depression, cancer)	AUC 0.60; 95% CI 0.50–0.71
Admission APACHE II	Single APACHE II score at admission	AUC 0.64; 95% CI 0.52–0.76
Admission SOFA	Single SOFA score at admission	AUC 0.63; 95% CI 0.51–0.76
Worst SOFA	Worst (most severe) SOFA score during ICU stay	AUC 0.71; 95% CI 0.61–0.79
Discharge SOFA	Single SOFA score at ICU discharge	AUC 0.56; 95% CI 0.45–0.67
Primary model (cumulative organ dysfunction)	No. of organ dysfunctions (sum of binary variables for delirium, hypoxemia, hypotension, AKI and liver injury with values from 0 to 5)	AUC 0.83; 95% CI 0.75–0.91 optimism-corrected AUC 0.82
Exploratory model (forward-selected three-variable)	Duration of index surgery, AKI during ICU, vasopressor-free days until day 30	AUC 0.87; 95% CI 0.79–0.95 optimism-corrected AUC 0.83

BMI, body mass index; CKD, chronic kidney disease; COPD, chronic obstructive pulmonary disease

A logistic regression model with all five organ dysfunctions entered as separate binary variables yielded an AUC of 0.83 (95% CI 0.75–0.91), comparable to the unweighted organ dysfunction count (AUC 0.82), supporting the pragmatic equal-weighting approach.

Findings were consistent across different specifications of the organ dysfunction burden (continuous and categorized) as well as different PICS definitions. Using the less specific PICS definition of impairment in any one dimension, 63 patients (40%) had PICS at 12 months. The use of this definition negatively impacted the performance of all prediction models. Similarly, analysis of each PICS component individually yielded poorer associations. Results of sensitivity analyses are summarized in Supplementary Table 1. Overall, across univariable and multivariable analyses, the duration, and complexity of the ICU course, summarized by organ dysfunction burden and related hemodynamic/organ-support measures, consistently associated with 12-month-prevalence of PICS. The strongest associations were observed for the primary a priori defined model of organ dysfunction burden and the specificity-focused PICS definition requiring impairment in at least two domains.

## Discussion

In this prospective cohort of ICU survivors, patients’ risk of suffering from PICS at 12 months after ICU discharge was significantly associated with the complexity of the acute critical illness. No statistical association was observed with baseline demographic or comorbidity profiles. Patients with PICS had longer ICU stays, fewer organ-support-free days, and more frequent organ dysfunctions and complications. A prespecified organ dysfunction metric showed a clear dose-response relationship with PICS and provided good discrimination (AUC 0.83; optimism-corrected 0.82), outperforming several established severity scores and baseline patient characteristics. A notable inflection in PICS risk was observed at a count of ≥ 3 organ dysfunctions (Fig. 2). Clinically, this or similar thresholds may in future serve as a pragmatic trigger for prioritizing intensive post-ICU follow-up, such as structured PICS screening and initiation of support. However, this threshold is data-derived from a single cohort and requires external validation before clinical implementation. In an exploratory multivariable model constrained to three variables, longer surgical duration, incidence of AKI, and fewer vasopressor-free days were independently associated with PICS and yielded an AUC of 0.87, but this apparent advantage largely disappeared after bootstrap optimism-correction (optimism-corrected AUC 0.83). Although single assessments of organ dysfunction (like admission SOFA or APACHE scores) do not predict long-term outcomes well, factors that add up to cumulative pathophysiological burden over the disease course do. Taken together, these findings support the hypothesis that the total in-ICU physiological insult may be a primary driver of long-term morbidity after critical illness.

### Relationship to prior work

Earlier studies of PICS outcomes were at times limited by insufficient ICU data, relying solely on variables like severity scores [11]. Our analysis enables us to corroborate and extend prior findings by including detailed individual-level data from ICU cases on the one hand, and standardized outcome assessment on the other, leading to an easily implemented metric like the organ dysfunction count. The absence of an association between baseline demographics and common comorbidities with PICS in our cohort strengthens the case that in- and peri-ICU trajectories, rather than fixed premorbid traits, dominate long-term outcomes in critically ill patients [14, 17, 18, 28–30]. Our approach adds to the available evidence by quantifying the breadth of physiological injury of critical illness as an organ dysfunction count and by demonstrating this metric’s discriminative ability for the occurrence of PICS. It is of note that the choice of PICS definition materially affects discrimination. Under the less restrictive ≥ 1 domain definition, AUC values were lower, indicating that the breadth of organ dysfunction is a stronger discriminator of multi-domain impairment than of any single isolated impairment. This likely reflects the greater etiological heterogeneity of single-domain impairment and its weaker specificity for ICU-acquired morbidity. Readers should interpret our reported AUCs in light of this definition-dependence, and future studies should examine whether the choice of PICS definition, and the precise way constructs are defined, influences the clinical utility of risk stratification tools.

### Interpretation and biological plausibility

Clinical intuition suggests that baseline physiological reserve influences susceptibility to organ dysfunction during the ICU stay, raising the question of whether the organ dysfunction count is indeed an independent driver of PICS or only a mediator through which baseline vulnerability exerts its effect. In our cohort, a baseline-only model showed no discriminative value (AUC 0.60; *p* = 0.72), and adding baseline factors to the organ dysfunction count did not alter discrimination (combined AUC 0.82, identical to the organ dysfunction count alone), suggesting that in this surgical ICU population, baseline characteristics contribute minimally to PICS risk beyond their potential influence on the ICU course itself. However, frailty was not formally assessed, and unmeasured baseline vulnerability could confound the observed associations. Future studies incorporating validated frailty instruments should examine whether the organ dysfunction count mediates the relationship between frailty and PICS, or whether it represents an independent risk signal. The unweighted organ dysfunction count assigns equal value to each affected organ system, despite heterogeneity in individual associations with PICS. A sensitivity analysis entering all five organ systems as separate predictors yielded an AUC of 0.83, comparable to the unweighted count. AKI and delirium were independently significant, consistent with their known associations with long-term impairment. Despite this heterogeneity, the equivalent AUC supports the pragmatic equal-weighting approach: The simple count captures the discriminative information. A weighted score derived from observed effect sizes would likely ultimately improve discrimination, but the size of our cohort was too small to reliably test this, and external validation with larger cohorts is needed. The observed pattern supports the case for the existence of a dose–response relationship: greater and more sustained organ dysfunction during the ICU stay is associated with higher likelihood of having PICS at one year. The fact that variables like the SOFA score performed worse is likely due to their lack of discriminatory power for acute organ injury in dimensions like cognition (SOFA measures GCS as opposed to specifically looking at delirium) and kidney injury (SOFA only looks at absolute creatinine values, not at trajectories). The graduated pattern of SOFA performance across timepoints is instructive: admission SOFA (AUC 0.63), most-severe SOFA (AUC 0.71), and discharge SOFA (AUC 0.56) all capture organ dysfunction at a single moment. The most-severe SOFA performs best among these because it captures peak illness, yet it still falls short of the cumulative count (AUC 0.83). The discharge SOFA performs worst because by ICU discharge, organ dysfunctions have largely resolved and the remaining score variance is minimal–the very information that matters for long-term outcomes (i.e., what the patient endured) is no longer visible in the score. While the strength and generalizability of our findings is limited by the modest cohort characteristics, this study’s results suggest that organ dysfunctions like AKI–markers of systemic inflammation, endothelial injury, and neurohumoral dysregulation [15, 16] (pathways that are plausibly linked to chronic neurocognitive and physical impairment) –may in the future be used to predict long-lasting multi-domain dysfunction. Fewer vasopressor-free days likely reflect prolonged hemodynamic instability and tissue hypoperfusion, which have been associated with neuromuscular and cognitive sequelae [19, 20]. The fact that a simple organ dysfunction count performed nearly as well as a more complex model suggests that the shared signal is the overall burden of organ dysfunction rather than a single organ system. This parsimony may in the future prove to be advantageous for transportability and clinical use, helping to guide follow-up planning and resource allocation after critical illness. The inclusion of 'duration of index surgery’ as a predictor in the exploratory model is inherently specific to surgical populations and would not be applicable to medical ICU cohorts. Future validation studies should include non-surgical ICU populations. Finally, AKI is both a component of the five-item organ dysfunction count (primary model) and the strongest individual predictor in the exploratory three-variable model. As a result, the two models share predictive information, and the incremental optimism-corrected AUC gain from 0.82 to 0.83 cannot be interpreted as entirely independent additional discrimination. The minimal AUC difference (0.01 after optimism-correction) likely reflects partial overlap in the captured risk signal rather than a fully independent improvement. This further reinforces our conclusion that the simpler organ dysfunction count captures much of the relevant information.

### Clinical implications

Despite its exploratory nature, two findings from our study are immediately actionable. First, a pragmatic risk signal (the number of organ dysfunctions) showed a strong gradient of PICS risk with good discrimination. This could be used for early identification of patients at high risk to prioritize post-ICU follow-up and rehabilitation resources. Because any single threshold identified on the same data is optimistic, such a rule should be validated externally before implementation. Second, the variables selected in the multivariable model (AKI, surgery duration, vasopressor-free days) as well as the other identified factors point to modifiable processes of care (hemodynamic optimization, kidney-protective strategies, infection control, sedation and delirium prevention) that may reduce downstream morbidity. While our observational design does in no way establish causal links, the consistency of directions across measures supports preventive strategies that target organ protection and instability mitigation during ICU care. In both unadjusted and adjusted analyses, the organ dysfunction count was inversely associated with EQ-5D-5L index at 12 months, indicating that the breadth of organ system involvement during the ICU stay is relevant to patient-reported quality of life beyond the binary PICS classification [31].

### Limitations

Several limitations warrant caution. First, this was a single-center cohort of only surgical patients with a modest sample, which limits precision and generalizability. Second, many ICU variables are interrelated (e.g., severity scores, organ dysfunction, support-free days, and length of stay), raising potential for collinearity and complicating attribution. Third, outcome ascertainment at 12 months was restricted to survivors; survivor and selection bias are unfortunately an inevitable consequence of the study design, and missingness of follow-up can distort associations. The attrition between screening and enrolment introduces potential selection bias. Patients who died before Day 90 represent the most severely ill subgroup and were necessarily excluded. Patients who could not be contacted or who declined participation may differ systematically from study participants in unmeasured ways. Ethics approval for this study did not permit detailed reporting of characteristics for patients who did not provide informed consent. The breadth of organ dysfunction that we found to be associated with PICS is also a driver of mortality. Our findings apply exclusively to survivors of the acute phase who reach the 12-month assessment. These considerations limit the generalizability of our prevalence estimates, though the internal associations between organ dysfunction count and PICS within the enrolled cohort remain interpretable. Fourth, even though we provide detailed data on pre-ICU comorbidities including psychological impairment, and we attempted to reconstruct baseline status in conversation with our patients, we lacked formal baseline quality-of-life measures, and premorbid function or frailty could confound some associations. The absence of formal pre-ICU baseline assessments is an important limitation. Some impairments classified as PICS at 12 months may have been pre-existing rather than acquired during or after the ICU stay. Future studies should incorporate validated pre-ICU functional assessments, ideally using preadmission evaluation, for example in surgical patients, or, where this is not possible, proxy-reported instruments administered early in the ICU stay. The organ dysfunction count captures only the number of affected organ systems (breadth) and does not account for the severity or duration of dysfunction within each system. Future studies should examine whether incorporating graded severity or duration of individual organ dysfunctions improves discrimination for PICS. In addition, our organ dysfunction definitions are based on individually selected criteria. Although each criterion was chosen for its clinical relevance and sensitivity for the specific organ system in the surgical ICU setting, the heterogeneity of these definitions represents a methodological trade-off, and results have to be interpreted in the context of the specific definitions. Finally, measurement limitations (e.g. potential misclassification of delirium or infections) may introduce noise that generally biases associations toward the null.

### Future directions

External validation of the hypothesis of organ dysfunction predicting PICS risk should be prioritized. Multicenter cohorts with larger samples can test whether such an approach adds value and whether its performance holds across case-mix and practice patterns. Future prospective work should also examine competing outcomes (death vs PICS) as well as severity of dysfunction in more detail. Finally, studies examining modifiable processes of care, like pragmatic interventional studies targeting protection of kidney function, hemodynamic stability, delirium prevention and interventions targeting nutrition and early mobilization are needed to evaluate whether attenuating organ dysfunction burden during the ICU stay reduces downstream PICS.

## Conclusions

In this single-center cohort of surgical ICU survivors, PICS at one year was more closely associated with the breadth of organ dysfunction during the ICU course than with baseline demographics or comorbidities. A simple organ dysfunction count captured much of the relevant risk signal and may provide a practical foundation for risk stratification at ICU discharge. These findings are hypothesis-generating and emphasize that mitigating organ dysfunction and hemodynamic instability during the acute phase of critical illness may be key to reducing long-term morbidity. Future multicenter studies with larger sample sizes are needed to validate these results and explore whether targeted organ protection and rehabilitation strategies can prevent the downstream burden of PICS.

## Supplementary Information

Supplementary Material 1.

## Data Availability

Due to ethical and legal restrictions (GDPR and consent limitations), potentially identifying data cannot be made publicly available. De-identified data are available upon reasonable request from the authors.

## Abbreviations

AKI: Acute kidney injury
APACHE: Acute physiology and chronic health evaluation
ARDS: Acute respiratory distress syndrome
AUC: Area under the receiver operating characteristic curve
BCa: Bias-corrected and accelerated
BMI: Body mass index
CAM-ICU: Confusion assessment method for the intensive care unit
CI: Confidence interval
CKD: Chronic kidney disease
COPD: Chronic obstructive pulmonary disease
ECMO: Extracorporeal membrane oxygenation
EQ-5D-5L: European quality of Life 5 dimensions 5 levels
GAD-7: Generalized anxiety disorder-7
GCS: Glasgow coma scale
ICU: Intensive care unit
IQR: Interquartile range
KDIGO: Kidney disease: improving global outcomes
MAP: Mean arterial pressure
OR: Odds ratio
PCL-5: PTSD checklist for DSM-5
PHQ-8: Patient health questionnaire-8
PICS: Post-intensive care syndrome
PTSD: Post-traumatic stress disorder
SD: Standard deviation
SOFA: Sequential organ failure assessment
