# Supplementary material for: Association between organ dysfunction during ICU stay and post-intensive care syndrome at 1 year after ICU discharge: a prospective cohort study
Source: J Intensive Care. 2026 May 28;14:48. doi: 10.1186/s40560-026-00885-4 (PMC13220465; doi:10.1186/s40560-026-00885-4)
Supplement: Supplementary file 1 — Supplementary Material 1. [file 40560_2026_885_MOESM1_ESM.docx]

**Supplementary Table 1:** Sensitivity Analyses using different PICS definitions and individual components

| **Variable** | **Bootstrap Inclusion Frequency** |
| --- | --- |
| AKI (yes/no) | 92% |
| Vasopressor-free days (0–30) | 69% |
| Duration of index surgery (min) | 60% |
| Delirium | 27% |
| Hypoxemia | 21% |
| Hemodynamic instability | 18% |
| Acute liver injury | 14% |
| ICU-acquired infection | 11% |

**Supplementary Table 2:** Bootstrap Inclusion Frequencies

| **Characteristic** | **0–2 Dysfunctions (n=89)** | **≥3 Dysfunctions (n=70)** |
| --- | --- | --- |
| Age, mean (SD) | 56 (17.5) | 62 (13.0) |
| Male sex, n (%) | 64 (71.9%) | 52 (74.3%) |
| BMI, mean (SD) | 27.8 (6.4) | 27.8 (5.3) |
| Hypertension, n (%) | 49 (55.1%) | 44 (62.9%) |
| Diabetes, n (%) | 17 (19.1%) | 16 (22.9%) |
| Depression, n (%) | 7 (7.9%) | 7 (10%) |
| CKD, n (%) | 5 (5.6%) | 6 (8.6%) |
| COPD, n (%) | 5 (5.6%) | 7 (10%) |
| Solid tumor, n (%) | 17 (19.1%) | 12 (17.1%) |
| **PICS at 12 months, n (%)** | **8 (9.0%)** | **41 (58.6%)** |

**Supplementary Table 3:** Characteristics by Organ Dysfunction Count

**
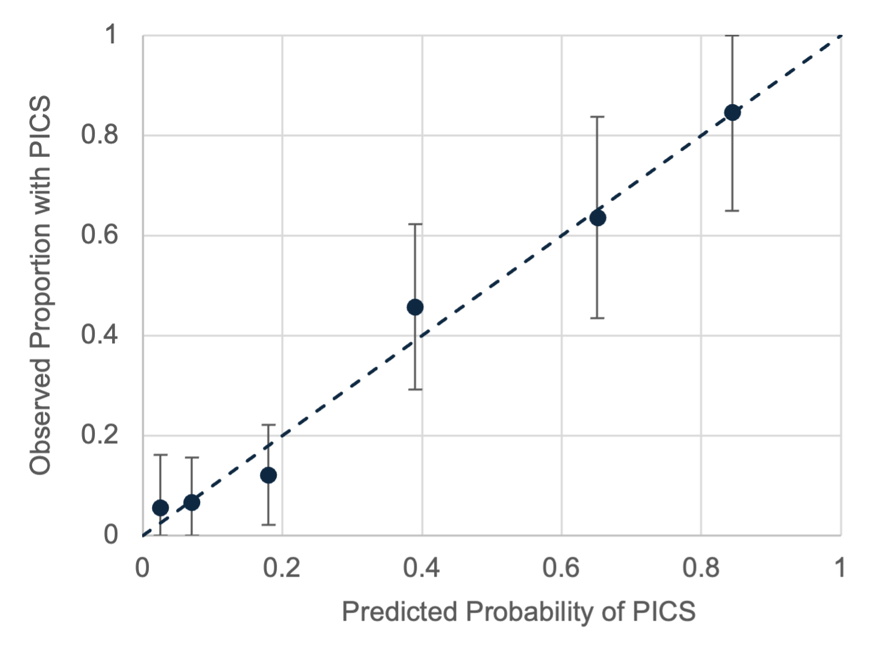
**

**Supplementary Figure 1:** Calibration plot for the primary organ dysfunction count model. Each point represents one level of the organ dysfunction count (0–5). The x-axis shows the model-predicted probability of PICS; the y-axis shows the observed proportion. Error bars represent 95% confidence intervals. The dashed line represents perfect calibration. Optimism-corrected calibration slope = 0.96; intercept = -0.02.


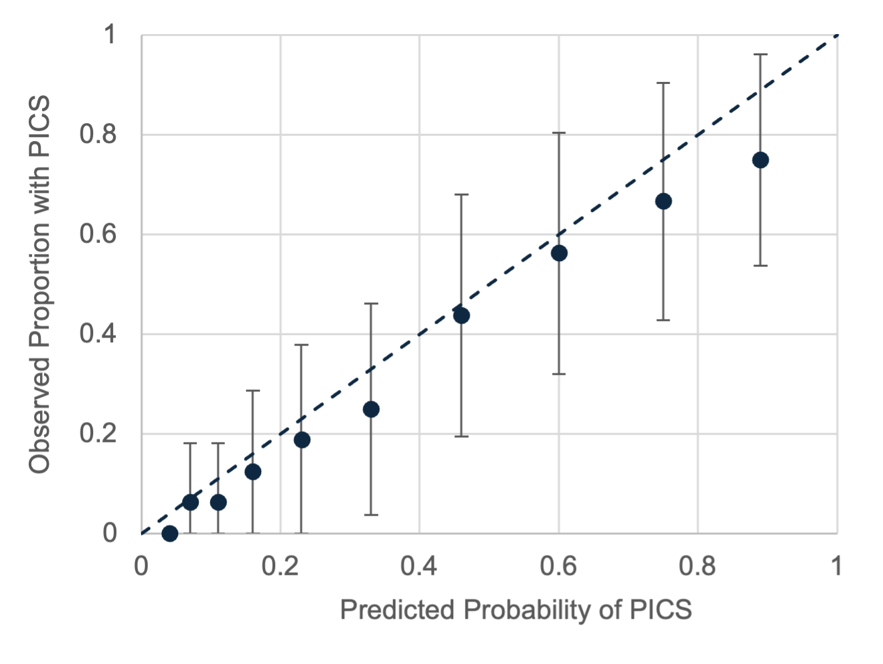


**Supplementary Figure 2:** Calibration plot for the exploratory three-variable model. Patients grouped into deciles of predicted risk. Optimism-corrected calibration slope = 0.80; intercept = 0.07. Deviation from the diagonal indicates overfitting, consistent with the borderline Hosmer-Lemeshow result (p = 0.049).
